# Comprehensive molecular characterization of lung tumors implicates AKT and MYC signaling in adenocarcinoma to squamous cell transdifferentiation

**DOI:** 10.1186/s13045-021-01186-z

**Published:** 2021-10-16

**Authors:** Alvaro Quintanal-Villalonga, Hirokazu Taniguchi, Yingqian A. Zhan, Maysun M. Hasan, Shweta S. Chavan, Fanli Meng, Fathema Uddin, Viola Allaj, Parvathy Manoj, Nisargbhai S. Shah, Joseph M. Chan, Metamia Ciampricotti, Andrew Chow, Michael Offin, Jordana Ray-Kirton, Jacklynn D. Egger, Umesh K. Bhanot, Irina Linkov, Marina Asher, Michael H. Roehrl, Katia Ventura, Juan Qiu, Elisa de Stanchina, Jason C. Chang, Natasha Rekhtman, Brian Houck-Loomis, Richard P. Koche, Helena A. Yu, Triparna Sen, Charles M. Rudin

**Affiliations:** 1 Department of Medicine, Thoracic Oncology Service, Memorial Sloan Kettering Cancer Center, 408 East 69th Street, ZRC-1731, New York, NY 10021 USA; 2 Center for Epigenetics Research, Memorial Sloan Kettering Cancer Center, New York, NY 10065 USA; 3 Sloan Kettering Institute, Memorial Sloan Kettering Cancer Center, New York, NY USA; 4 Marie-Josée and Henry R. Kravis Center for Molecular Oncology, Memorial Sloan Kettering Cancer Center, New York, NY USA; 5 Program for Computational and Systems Biology, Sloan Kettering Institute, Memorial Sloan Kettering Cancer Center, New York, NY USA; 6 Parker Institute for Cancer Immunotherapy, Memorial Sloan Kettering Cancer Center, New York, NY USA; 7 Precision Pathology Center, Memorial Sloan Kettering Cancer Center, New York, NY USA; 8 Department of Pathology, Memorial Sloan Kettering Cancer Center, New York, NY USA; 9 Human Oncology and Pathogenesis Program, Memorial Sloan Kettering Cancer Center, New York, NY USA; 10 Antitumor Assessment Core, Memorial Sloan Kettering Cancer Center, New York, NY 10065 USA; 11 Weill Cornell Medical College, 1275 York Avenue, New York, NY 10065 USA; 12 Molecular Pharmacology Program, Memorial Sloan Kettering Cancer Center, 1275 York Avenue, New York, NY 10065 USA

**Keywords:** Lineage plasticity, Squamous transdifferentiation, Treatment resistance, Targeted therapy

## Abstract

**Background:**

Lineage plasticity, the ability to transdifferentiate among distinct phenotypic identities, facilitates therapeutic resistance in cancer. In lung adenocarcinomas (LUADs), this phenomenon includes small cell and squamous cell (LUSC) histologic transformation in the context of acquired resistance to targeted inhibition of driver mutations. LUAD-to-LUSC transdifferentiation, occurring in up to 9% of *EGFR*-mutant patients relapsed on osimertinib, is associated with notably poor prognosis. We hypothesized that multi-parameter profiling of the components of mixed histology (LUAD/LUSC) tumors could provide insight into factors licensing lineage plasticity between these histologies.

**Methods:**

We performed genomic, epigenomics, transcriptomics and protein analyses of microdissected LUAD and LUSC components from mixed histology tumors, pre-/post-transformation tumors and reference non-transformed LUAD and LUSC samples. We validated our findings through genetic manipulation of preclinical models in vitro and in vivo and performed patient-derived xenograft (PDX) treatments to validate potential therapeutic targets in a LUAD PDX model acquiring LUSC features after osimertinib treatment.

**Results:**

Our data suggest that LUSC transdifferentiation is primarily driven by transcriptional reprogramming rather than mutational events. We observed consistent relative upregulation of PI3K/AKT, MYC and PRC2 pathway genes. Concurrent activation of PI3K/AKT and MYC induced squamous features in *EGFR*-mutant LUAD preclinical models. Pharmacologic inhibition of EZH1/2 in combination with osimertinib prevented relapse with squamous-features in an EGFR-mutant patient-derived xenograft model, and inhibition of EZH1/2 or PI3K/AKT signaling re-sensitized resistant squamous-like tumors to osimertinib.

**Conclusions:**

Our findings provide the first comprehensive molecular characterization of LUSC transdifferentiation, suggesting putative drivers and potential therapeutic targets to constrain or prevent lineage plasticity.

**Supplementary Information:**

The online version contains supplementary material available at 10.1186/s13045-021-01186-z.

## Background

Lineage plasticity, the ability of cells to transdifferentiate from one committed fate to a distinct developmental lineage, has been implicated as a driver of resistance to targeted therapies in multiple cancer types [1]. In patients with lung adenocarcinoma (LUAD), the advent of potent and selective targeted inhibitors of primary driver mutations, and the increased practice of tumor re-biopsy at the time of treatment relapse, has led to increasing recognition of lineage plasticity as a common mechanism of acquired resistance and treatment failure. The first example of this phenomenon to be characterized in lung cancer was neuroendocrine transformation of *EGFR*-mutant LUADs to tumors resembling small cell lung cancer [2, 3]. Such tumors retain the *EGFR* driver mutation but lose dependence on EGFR signaling for survival and proliferation. More recently, histologic transdifferentiation of LUAD to squamous carcinoma of the lung (LUSC) has also been observed in association with acquired resistance to highly active targeted therapies [4–6]. Interestingly, even if targeted agents may promote transdifferentiation, the existence of untreated adenosquamous tumors containing areas of LUAD and LUSC showing shared driver alterations in both histological components [7, 8] suggests that this lineage plasticity phenomenon may occur in the absence of the selective pressure exerted by treatment. Mixed histology non-small cell lung cancer has a remarkably poor prognosis across stage, significantly worse than either LUAD or LUSC [9]. A better understanding of potential drivers of histologic transdifferentiation in lung cancer is desperately needed to define more effective therapeutic strategies for these patients.

The primary drivers of LUAD to LUSC transdifferentiation have not been defined in human tumors. Lineage plasticity between LUAD and LUSC histologies has been observed in a murine model of *Kras*-mutant LUAD that transdifferentiated to LUSC upon *Lkb1* loss [10], but *STK11* (encoding human LKB1) loss-of-function mutations and deletions are among the most common somatic events in human LUAD [11]. Due to the sparseness of well-annotated matched pre- and post-clinical specimens and the absence of representative preclinical models, little is known about the molecular alterations that facilitate LUSC transdifferentiation.

Adenosquamous carcinomas containing discrete areas of LUAD and LUSC histology amenable for physical isolation for independent molecular analyses may provide insight into lineage plasticity along this axis. The occurrence of common driver mutations in both histologic components in such cases [7, 8] suggests a recent divergence in phenotypic commitment to adenomatous vs. squamous lineages. We hypothesized that defining common genetic and epigenetic differences between LUAD and LUSC components in the constrained environment of these mixed histology tumors could define candidate determinants of LUAD to LUSC transdifferentiation, which could be subsequently validated in manipulable models. In this study, we examined lineage plasticity between LUAD and LUSC by integrated genomic, epigenomic, transcriptomic and protein analyses of (1) mixed LUAD/LUSC tumors with discrete areas of each histology and (2) pre- and post-transdifferentiation specimens, including matched pairs. We then interrogated the effects of candidate drivers in preclinical models of human LUAD. Our findings provide the first comprehensive molecular characterization of LUSC transdifferentiation, nominating key drivers and potential therapeutic targets to prevent or treat this mechanism of tumor escape from targeted therapies.

## Methods

### Clinical specimens

11 Formalin-fixed paraffin-embedded (FFPE) tumors with combined LUAD and LUSC histology were identified, from which independent isolation of both histological components was possible (*N* = 11, Additional file 1: Table S1). As the components of these mixed histology tumors are not temporally ordered, we refer to the component parts of these mixed histology tumors as “T-LUAD” and “T-LUSC” with the T referring to histologic transformation, without presumption of directionality. We identified an additional 4 pre-transformation LUAD and 7 post-transformation LUSC cases for which tissue material was available (Additional file 1: Table S1). As controls, we included a group of never-transformed LUADs (*N* = 15) and a set of de novo LUSC samples (*N* = 11) (Additional file 1: Tables S2,3). All study subjects had provided signed informed consent for biospecimen analyses under an institutional review board-approved protocol.

### Tissue isolation

For microdissection, hematoxylin and eosin (H&E)-stained FFPE tumor slides of tumors with combined LUAD/LUSC were evaluated by a pathologist. Multiple FFPE blocks of each tumor were reviewed, with the aim of selecting areas containing exclusively the LUAD or the LUSC component. Where individual slides with pure components were not available, slides containing both histologic components with complete physical separation were selected. Between 10 and 20 unstained sections (USS) at 10 μm prepared on uncharged slides from corresponding FFPE blocks were used for microdissection of each case. Every 10 sections, an additional section was stained with H&E for confirmation of histology. The areas corresponding to each histological component on the initial H&E were dissected using a clean blade and the tissue collected in 0.5-ml nuclease-free tubes for nucleic acid extraction. Alternatively, 1.0–1.5-mm core punches were made from LUAD and LUSC areas on the FFPE blocks and placed in 0.5-ml nuclease-free tubes for nucleic acid extraction, exclusively in cases where each histologic component was located in a different block and where no histologic cross-contamination was confirmed by pathological review.

### DNA extraction

FFPE tissue was deparaffinized using heat treatment (90 °C for 10’ in 480 μL PBS and 20 μL 10% Tween 20), centrifugation (10,000 × *g* for 15’) and ice chill. Paraffin and supernatant were removed, and the pellet was washed with 1 mL 100% EtOH followed by an incubation overnight in 400 µl 1 M NaSCN for rehydration and impurity removal. Tissues were subsequently digested with 40 µl Proteinase K (600 mAU/ml) in 360 µl Buffer ATL at 55 °C. DNA isolation proceeded with the DNeasy Blood & Tissue Kit (QIAGEN catalog #69504) according to the manufacturer’s protocol modified by replacing AW2 buffer with 80% ethanol. DNA was eluted in 0.5X Buffer AE.

### RNA/DNA dual extraction from FFPE tissue

FFPE sections were deparaffinized in mineral oil. Briefly, 800µL mineral oil (Fisher Scientific, #AC415080010) and 180µL Buffer PKD were mixed with the sections, Proteinase K was added for tissue digestion, and the sample was incubated at 56 °C for 15 min. Phase separation was encouraged with centrifugation, and the aqueous phase was chilled 3 min to precipitate RNA. After centrifugation for 15 min at 20,000* g,* RNA-containing supernatant was removed for extraction, while DNA remained in the pellet. Nucleic acids were subsequently extracted using the AllPrep DNA/RNA Mini Kit (QIAGEN, #80204) according to the manufacturer’s instructions. RNA was eluted in nuclease-free water and DNA in 0.5X Buffer ATE.

### Whole exome sequencing

For DNA samples, after PicoGreen quantification and quality control by Agilent BioAnalyzer, 100–500 ng of DNA were used to prepare libraries using the KAPA Hyper Prep Kit (Kapa Biosystems KK8504) with 8 cycles of PCR. After sample barcoding, 100 ng of library was captured by hybridization using the xGen Exome Research Panel v1.0 (IDT) according to the manufacturer’s protocol. PCR amplification of the post-capture libraries was carried out for 12 cycles.

For DNA library samples, after PicoGreen quantification and quality control by Agilent BioAnalyzer, 100 ng of library transferred from the DMP was captured by hybridization using the xGen Exome Research Panel v1.0 (IDT) according to the manufacturer’s protocol. PCR amplification of the post-capture libraries was carried out for 8 cycles.

Samples were run on a HiSeq 4000 in a 100 bp/100 bp paired end run, using the HiSeq 3000/4000 SBS Kit (Illumina). Normal and tumor samples had a median target coverage of 87X and 108X, respectively.

### Whole exome analysis

We used a comprehensive in-house WES pipeline TEMPO—time-efficient mutational profiling in oncology (https://github.com/mskcc/tempo & https://ccstempo.netlify.app) that performs alignment using BWA-mem algorithm followed by mutation calling using Strelka2 and Mutect2 variant callers. The combined, annotated and filtered variant calls were used for downstream analysis. Details of the variant call processing are described at https://ccstempo.netlify.com/variant-annotation-and-filtering.html#somatic-snvs-and-indels and are previously described as well [12]. Copy number analysis was performed with FACETS (https://github.com/mskcc/facets), processed using facets-suite (https://github.com/mskcc/facets-suite), and manual reviewed and refitted using facets-preview (https://github.com/taylor-lab/facets-preview). To delineate mutational processes driving the acquisition of somatic alterations, mutational signatures were decomposed for all tumor samples that had a minimum of 5 single-nucleotide somatic mutations using the R package mutation signatures (https://github.com/mskcc/mutation-signatures). Further, a given signature was considered to be ‘dominant’ if the proportion of mutations contributing to the signature was at least 20% of all mutations detected in the sample.

Purity, ploidy, tumor mutational burden (TMB), genome doubling and cancer cell fractions for all mutations in all specimens were inferred from sequencing data. We estimated neoantigen load by taking the number of variant estimated to having strong class I MHC binding affinity by NetMHC 4.0 [13] and normalizing it by the TMB. We summarized the top occurring somatic variants located on cancer genes in an oncoprint using the R package *ComplexHeatmaps* version 2.0.0 (https://github.com/jokergoo/ComplexHeatmap) [14]. Cancer genes were genes defined as “OncoKB Annotated” on the Cancer Gene List (downloaded in June 2020, https://www.oncokb.org/cancerGenes). All other plots for this analysis were created using *ggplot2* version 3.3.2 (https://github.com/tidyverse/ggplot2).

### Comparison to TCGA

Somatic mutations and copy number alterations (CNAs) found in cancer genes in our T-LUAD samples were compared to those found in The Cancer Genome Atlas Lung Adenocarcinoma (TCGA-LUAD) cohort using a Fisher exact test. The mutations from TCGA-LUAD [15] were extracted using the R package TCGA mutations (https://github.com/PoisonAlien/TCGAmutations) and tested against our cohort mutations with maftools v.2.0.16 (https://github.com/PoisonAlien/maftools) [16]. Separately, a Fisher exact test was used to identify significant CNAs by comparing the number of samples with amplifications and deletions on particular genes in TCGA-LUAD, extracted from CbioPortal [17, 18], to the number of samples with gene level CNAs in our cohort. For both mutations and CNAs, genes with *p* < 0.05 were considered differentially altered. The results were summarized in a volcano plot using the R packages, EnhancedVolcano version 1.7.4 (https://github.com/kevinblighe/EnhancedVolcano) and ggplot.

### Methylation sequencing

After PicoGreen quantification (ThermoFisher, #P11496) and quality control by Agilent BioAnalyzer, 170–750 ng of genomic DNA was sheared using a LE220-plus Focused-ultrasonicator (Covaris, #500569). Samples were cleaned using Sample Purification Beads from the TruSeq Methyl Capture EPIC LT Library Prep Kit (Illumina, #FC-151-1002) according to the manufacturer’s instructions with modifications. Briefly, samples were incubated for 5 min after addition of SPB, 50 µL RSB was added for resuspension, and resuspended samples were incubated for 2 min. Sequencing libraries were prepared using the KAPA Hyper Prep Kit (Kapa Biosystems KK8504) without PCR amplification. Post-ligation cleanup proceeded according to Illumina’s instructions with 110 µL Sample Purification Mix. After purification, 3–4 samples were pooled equimolar and methylome regions were captured using EPIC oligos. Capture pools were bisulfite converted and amplified with 11–12 cycles of PCR. Pools were sequenced on a NovaSeq 6000 or HiSeq 4000 in a 150/150 bp or 100 bp/100 bp paired end run, using the NovaSeq 6000 S4 Reagent Kit (300 Cycles) or HiSeq 3000/4000 SBS Kit (Illumina). The average number of read pairs per sample was 51 million.

### DNA methyl capture EPIC data processing

The Bismark pipeline [19] was adopted to map bisulfite-treated EPIC sequencing reads and determine cytosine methylation states. Trim Galore v0.6.4 was used to remove raw reads with low-quality (less than 20) and adapter sequences. The trimmed sequence reads were C(G) to T(A) converted and mapped to similarly converted reference human genome (hg19) [20] using default Bowtie 2 [21] settings within Bismark. Duplicated reads were discarded. The remaining alignments were then used for cytosine methylation calling by Bismark methylation extractor.

### Differential methylation analysis

Differentially methylated CpGs (DMCs) were identified using DSS R package [22, 23] on the basis of dispersion shrinkage followed by Wald statistical test for beta-binomial distributions. Any CpGs with FDR < 0.1 and methylation percentage difference greater than 10% were considered significant DMCs. Differentially methylated regions (DMRs) were subsequently called based on the DMCs. The called DMRs were required to satisfy the minimum length of 50bps and minimum 3 CpGs in the region; two neighboring DMRs were merged if less than 50bps apart, and significant CpGs were those that occupy at least 50% of all CpGs population in the called DMRs as default in DSS package. Pairwise comparisons were conducted for pre-transformation pre-transformation LUAD versus control LUAD, post-transformation LUSC versus de novo LUSC, and post-transformation LUSC versus pre-transformation LUAD. The DMRs were mapped to gene regions at promoters and gene bodies, and differential methylation levels were subsequently associated with differential gene expression values in selected pathways. In addition to pairwise comparisons, principal component analysis (PCA) and partial least square discriminant analysis (PLSDA) were also performed to classify samples into groups and identify influential CpGs using mixOmics R package [23].

### Motif enrichment analysis

Differential methylation may influence transcription factor (TF) binding. To identify overrepresented known TF motifs due to differential methylation for the post-transformation LUSC compared with pre-transformation LUAD, “findMotifsGenome.pl” from HOMER [24] was applied to DMCs (± 50bps) overlapping with gene promoter regions. DMCs regions with hyper- and hypo-methylation in LUSC were explored separately to show the effects from different methylation status. The significantly enriched TFs were defined as those with *q* value ≤ 0.1.

### RNA sequencing

Approximately 500 ng of FFPE RNA or 100 ng of fresh frozen RNA per sample was used for RNA library construction using the KAPA RNA Hyper library prep kit (Roche, Switzerland) per the manufacturer’s instructions with minor modifications. Customized adapters with unique molecular indexes (UMI) (Integrated DNA Technologies, US) and Sample-specific dual-indexes primers (Integrated DNA Technologies, US) were added to each library. The quantity of libraries was measured with Qubit (Thermo Fisher Scientific, US) and quality measured by TapStation Genomic DNA Assay (Agilent Technologies, US). Equal amounts of each RNA library (around 500 ng) were pooled for hybridization capture with IDT Whole Exome Panel V1 (Integrated DNA Technologies, US) using a customized capture protocol modified from NimbleGen SeqCap Target Enrichment system (Roche, Switzerland). The captured DNA libraries were then sequenced on an Illumina HiSeq4000 with paired end reads (2 Å ~ 100 bp), at 50millions reads/sample.

### RNASeq analysis

In-line UMI sequences were trimmed from the sequencing reads with Marianas (https://github.com/mskcc/Marianas) and aligned to human GRCh37 genome using STAR 2.7.0 (https://github.com/alexdobin/STAR) [25] with Ensembl v75 gene annotation. Hybrid selection-specific metrics and alignment metrics were calculated for the BAM files using CalculateHsMetrics and CollectRnaSeqMetrics, respectively, from Picard Toolkit (https://github.com/broadinstitute/picard) to determine the quality of the capture.

We quantified RNA-seq reads with Kallisto v.0.45.0 [26] to obtain transcript counts and abundances. Kallisto was run with 100 bootstrap samples, sequence-based bias correction and in strand-specific mode, which processed only the fragments where the first read in a pair is pseudoaligned to the reverse strand of a transcript. Differential gene expression analysis, principle component analysis and transcript per million (TPM) normalization by size factors were done from Kallisto output files using Sleuth v0.30.0 run in gene mode [27]. Differentially expressed genes were identified using the Wald test. Genes were marked significant if the False Discovery Rates, *q*, calculated using the Benjamini–Hochberg method, were less than 0.05, and *beta* (Sleuth-based estimation of log2 fold change) > 1.25, which approximately correlated with a log2 fold change of 2 in our data. The log of the normalized TPM values for selected significant genes was rescaled using a *z*-score transformation and plotted in a heatmap using the ComplexHeatmap Library in R.

### Pathway enrichment

Gene set enrichment analysis (GSEA) [28] was performed on full sets of gene expression data across the previously mentioned three comparisons. Genes were ranked on *p* value scores computed as − log10(*p* value) * (sign of beta). Gene set annotations were taken from Molecular Signatures Database (MSigDB v7.0.1) [28, 29]. The significance level of enrichment was evaluated using permutation test, and the *p* value was adjusted by Benjamini–Hochberg procedure. Any enriched gene sets with adjusted *p* value ≤ 0.1 were regarded as significant. This analysis was conducted using ClusterProfiler R package [30]. The enriched gene sets that are influenced by DMCs were selected, and pathway annotations concatenated manually to remove redundancy and achieve high level generality. When the pathway terms were merged, median enrichment score was taken as the new group enrichment score, p values were aggregated using Fisher’s method from the Aggregation R package [31], and core enrichment of genes was collapsed.

### LUSC subtyping

Wilkerson’s model [32] was used to predict LUSC subtypes by a nearest centroid classification algorithm. An expression heatmap on centroid genes was produced by ‘ComplexHeatmap’ library in R. Chi-square test was performed to detect the association between cell types and subtypes.

### Immunoblotting

Protein extraction and Western blot were performed as previously described [33] after quantification of protein extracts using the Bradford method (#5000205, Bio-Rad), running 10–30-ug aliquots in the gels. Western blot antibodies for Beta-catenin (#8480, Cell Signaling Technology), pAKT (#4060, Cell Signaling Technology), p40 (#67825, Cell Signaling Technology), EGFR (#4267, Cell Signaling Technology), EZH2 (#5246, Cell Signaling Technology), MYC (#13987, Cell Signaling Technology), pPRAS40 (#2997, Cell Signaling Technology), SOX2 (#3579, Cell Signaling Technology), Vinculin (#13901, Cell Signaling Technology) and actin (#3700, Cell Signaling Technology) were used. Immunohistochemistry antibodies for p40 (#AC13066A, BioCare), TTF-1 (#M3575, Dako), CK5/6 (#790-4554, Ventana) and MYC (#ab32072, Abcam) were used.

### Protein extraction from FFPE

Protein extraction from punches on the LUAD and LUSC components of combined histology FFPE blocks was performed following the instructions from the Qproteome FFPE Tissue Kit (#37623, Qiagen), using one punch per extraction.

### Phospho-kinase array

Protein samples were quantified with the Bradford method (#5000205, Bio-Rad), and 200-ug aliquots were used in the phospho-kinase array (#ARYC003C, R&D-Biotechne), which was performed using the manufacturer’s instructions. Quantification of spots was performed using the Image Studio software (Version 3.1, Li-Cor). Technical replicates (2 per array) per sample were averaged. Two-tailed Student’s T-test was performed on these values, comparing the T-LUAD and T-LUSC groups.

### Cell line transductions

Lx462 cell line was derived by PDX dissociation using the tumor dissociation kit (#130-95-929, Miltenyi) and GentleMACS Octo Dissociator with Heaters (Miltenyi, #130-096-427) as indicated by the manufacturer. Dissociated cells were seeded in RPMI 1640 10% FBS and expanded in culture. PC9 cell line was purchased from Millipore Sigma (#90071810-VL). Both cell lines were regularly tested for Mycoplasma and maintained in RPMI 1640 10% FBS. Lentiviruses were produced as previously described [34] with a MYC overexpression plasmid (#10674, Addgene) and a myrAKT overexpression plasmid, kindly provided by Dr. Witte [35]. Cell lines were transduced at high MOI as previously described [34] with overnight virus incubation.

### Xenografts and in vivo treatments

For cell line xenografts, 1 million cells were injected in a 1:1 mixture of PBS and Matrigel (#CB40234, Fisher) in the flanks of NOD.Cg-Prkdc < scid > Il2rg < tm1Wjl > /SzJ (NSG) mice. For patient-derived xenografts, tumor was dissociated as described above, and 1 million cells were injected in a 1:1 mixture of PBS and Matrigel (#CB40234, Fisher) in the flanks of NOD.Cg-Prkdc < scid > Il2rg < tm1Wjl > /SzJ (NSG) mice.

For treatments, 5–6 mice were engrafted per treatment arm (please see figure legends for details) until they reached 100–150 mm^3^. At that point, mice were randomized into groups and treated with either vehicle, osimertinib (25 mg/kg/day), ORS1 (100 mg/kg/day) or samotolisib (10 mg/kg/day), or combinations of osimertinib and ORS1, or of osimertinib and samotolisib, by oral gavage 5 days a week. Mice were killed when tumors reached ~ 1000 mm^3^ and fixed in formalin 10% O/N for paraffin embedding. Tumors and mice body weight were measured twice a week.

To generate osimertinib-resistant Lx462 tumors, these were treated with osimertinib in the previously mentioned conditions until relapse (~ 1000 mm^3^), when tumors were collected, dissociated and re-engrafted to continue osimertinib treatment. At second relapse on osimertinib, mice were considered resistant.

## Results

### Genomic characterization of LUSC transformation

**Fig. 1:**
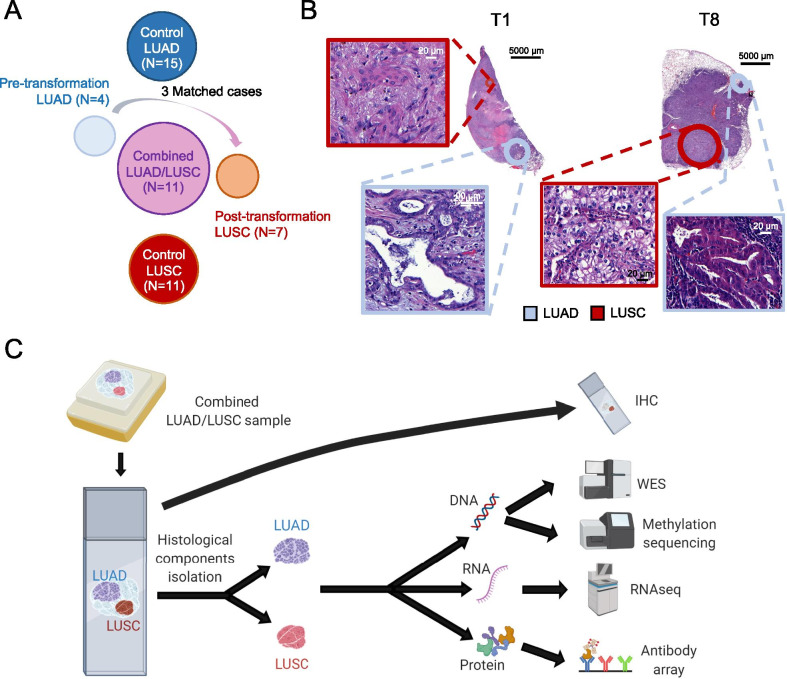
Multilayer molecular characterization of LUSC transformation. **a** Schematic composition of the cohort under study. **b** Illustrative H&E images of two adenosquamous samples with clear spatial isolation of the LUAD and LUSC components. **c** Schema showing the processing of adenosquamous samples for molecular analyses

We began our analysis by identifying eleven lung adenosquamous clinical specimens with clear spatial separation of the LUAD and LUSC components (Figs. 1a, b and Additional file 1: Table S1, Samples #1–11). We also identified pure histology pre-transdifferentiation LUADs (*N* = 4) and post-transdifferentiation LUSC (*N* = 7), including 3 matched cases (Fig. 1a and Additional file 1: Table S1 Samples #12–19), of squamous relapse on targeted therapy with known previous history of adenocarcinoma, where a common driver alteration was confirmed clinically by targeted sequencing. Control LUAD (*N* = 15) and LUSC (*N* = 11) samples with no history of histologic transitions were analyzed in parallel as an anchor for group comparisons made (Fig. 1a and Additional file 1: Tables S2,3). In the adenosquamous cases, LUAD and LUSC components were micro-dissected and subjected to independent genomic, methylomic, transcriptomic and protein analyses (Fig. 1c and Additional file 1: Table S4).

**Fig. 2:**
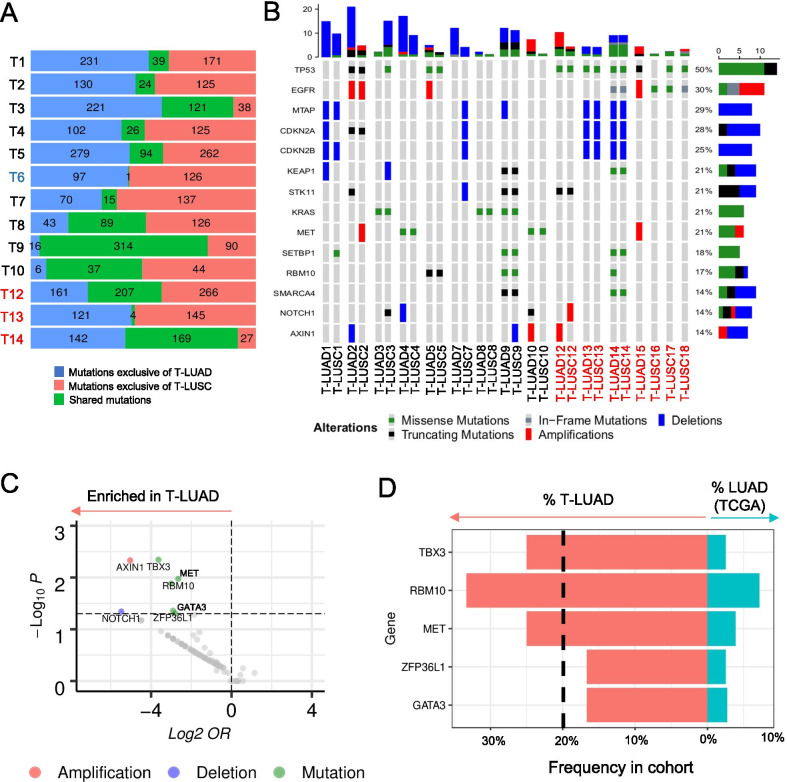
Genomic characterization of LUSC transformation. **a** Bar blot showing number of mutations occurring specifically in the T-LUAD (blue) and T-LUSC (red) components, and of mutations shared between these (green). **b** Oncoprint showing the most prevalent mutations and CNAs in the transformation samples. **c** Volcano plot showing enrichment (as shown by odds ratio, OR) of genomic alterations in our T-LUAD samples, compared to the TCGA LUAD samples, used as control cohort. **d** Barplot showing the percentage of cases harboring mutations in genes of interest in the T-LUAD (red) and TCGA LUAD (blue) cohorts. Samples IDs in black indicate that they come from a combined histology specimen where LUAD and LUSC components are genetically related. Samples IDs in blue indicate that they come from a combined histology specimen where LUAD and LUSC components are genetically unrelated. Samples IDs in red indicate that they come from a pre-/post-transformation specimen

Mixed histology tumors with discrete areas of LUAD and LUSC could reflect subclonal histologic divergence of a single parental clone, or alternatively could reflect independent oncogenic events arising in a patient at risk of lung cancer due to field carcinogenesis. To confirm a common clonal origin of both histological derivatives in these samples, we analyzed all paired samples for shared somatic mutations. Whole exome sequencing (WES) of LUAD and LUSC components from adenosquamous specimens (*N* = 11) and the matched pre- and post-transdifferentiation pairs (*N* = 3) revealed multiple shared mutations in all but one case (T6). Although low tumor purity of the LUAD component in this questionable case may explain this result (Additional file 2: Figure S1B), we elected to discard this case from further analyses (Fig. 2A). For one adenosquamous case (T11) with no WES data available, clonal relation of the LUAD and LUSC components was confirmed by detection of shared mutations in the RNAseq data (Additional file 2: Figure S1A). We refer to the genetically related samples hereafter as T-LUAD and T-LUSC with the T referring to histologic transdifferentiation, with no presumption of directionality. These results further support the existence of lineage plasticity in adenosquamous samples, consistent with previous reports [7, 36].

There were no consistent differences in sample purity (Additional file 2: Figure S1B), sample ploidy (Additional file 2: Figure S1C), or tumor mutational burden (Additional file 2: Figure S1D) between T-LUAD and T-LUSC samples. Analyses of mutational signatures revealed a predominance of smoking and aging signatures as expected, without consistent differences between T-LUAD and T-LUSC pairs (Additional file 2: Figure S1E). Frequently observed genomic alterations included mutation of *TP53* (9/16 cases) and *EGFR* (7/16 cases), and deletion of genes located in the 9q21 chromosome region including *MTAP* (5/16 cases), *CDKN2A* (4/16) and *CDKN2B* (4/16) (Fig. 2b). Four of 16 cases showed alterations of *STK11* (Fig. 2b), similar to the observed rate of 17% among over 6700 LUAD cases sequenced at our institution. None of these frequently altered genes were differentially mutated between matched T-LUAD and T-LUSC samples. These results do not suggest a particular genomic context in which LUSC transformation is strongly favored.

To further explore genetic alterations that might facilitate LUSC transdifferentiation, we performed enrichment analyses comparing our T-LUAD samples with TCGA LUADs as control (Figs. 2c, d and Additional file 2: Figure S1F). We focused only on those genomic alterations occurring in > 20% of T-LUAD due to the limited size of our cohort (Fig. 2d). We found significant enrichment of mutations in *TBX3* (25.0% T-LUAD versus 2.6% LUAD, *p*-value = 0.005), a transcriptional repressor involved in development and overexpressed in multiple cancer types, including squamous tumors [37, 38]; *MET* (25% T-LUAD versus 4.0% LUAD, *p*-value = 0.013), a receptor tyrosine kinase whose amplification confers resistance to EGFR inhibitors in *EGFR*-mutant LUADs [39] and is altered in 6% of LUSC(40); and *RBM10* (33.3% T-LUAD versus 7.3% LUAD, *p*-value = 0.011), an RNA binding protein and alternative splicing regulator, implicated as a tumor suppressor and frequently deleted or mutated in human cancers [41, 42] (Fig. 2d). These data nominate *TBX3, MET*, and *RBM10* alterations as molecular contexts that may facilitate transdifferentiation. Even if unlikely drivers, these alterations may potentially serve as predictors of LUSC transdifferentiation.

### T-LUSC is enriched in the secretory subtype, and retains transcriptomic and methylomic features of T-LUADs

**Fig. 3:**
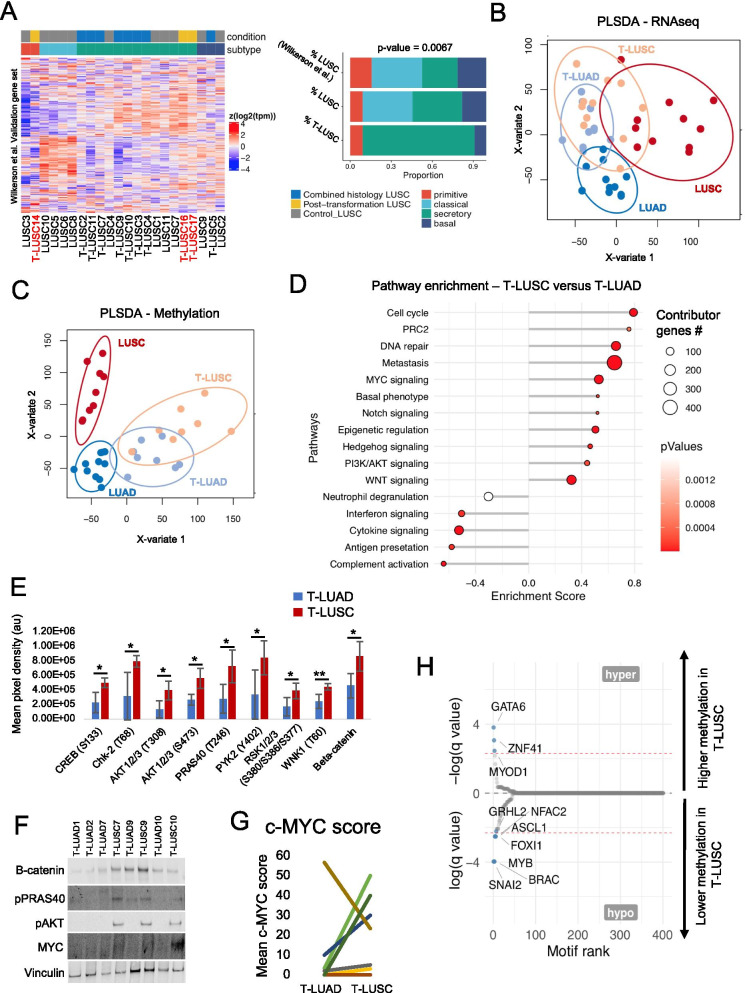
Transcriptomic, epigenomic and protein characterization of LUSC transformation. **a** Heatmap showing the expression of genes predictive of LUSC subtype in the LUSC and T-LUSC samples in our cohort (left) and bar plot indicating the percentage of each subtype present in our LUSC and T-LUSC cohorts, with comparison to Wilkerson et al.18. *p*-value was calculated using the Chi-Square test assessing the distribution differences between cohorts. PLSDA analyses on the transcriptome (**b**) and (**c**) methylome of T-LUAD and T-LUAD samples, and of control LUAD and SCLC samples. Circles delimiting each sample group were calculated with 90% interval of confidence. **d** Pathway enrichment analyses on DEGs of the T-LUSC versus T-LUAD comparison. **e** Bar plot showing differential expression of phosphorylated proteins involved in the AKT, Wnt and DNA damage response pathways, as determined by an antibody array on microdissected LUAD and LUSC tissue from adenosquamous clinical samples (see Additional file 1; Table S4). **f** Western blot showing expression/phosphorylation of proteins of interest on the same samples analyzed by protein array. **g** MYC protein expression levels (IHC score) on matched T-LUAD and T-LUSC components in adenosquamous samples. **h** Plot exhibiting differentially methylated transcription factor binding domains in T-LUSC versus T-LUAD. Sample IDs in black and red indicate that they come from a combined histology specimen or a pre-/post-transformation specimen, respectively. *p*-values legend: *p < 0.05, **p < 0.01

We next performed transcriptome (RNAseq) and methylation (EPIC) analyses of T-LUADs, T-LUSCs, control LUADs and LUSCs. LUSCs have been divided into four subtypes distinguished by distinct transcriptional profiles, termed classical, secretory, primitive and basal [32]. Subtype distribution in control LUSCs in our cohort essentially mirrored previously reported cohorts (Fig. 3a) [32]. However, subtype distribution among T-LUSCs was substantially skewed, with no classical subtype samples and a marked enrichment of the secretory subtype (*p* = 0.0067) (Fig. 3a and Additional file 2: Figure S2A). Notably, the transcriptional program of the secretory subtype is most closely aligned with that of LUAD [32].

To gain further insight into the commonalities and differences among our defined cohorts, we performed partial least squares discriminant analysis (PLSDA) on both the transcriptional and methylation datasets. PLSDA of the RNAseq data showed distinct clustering of control LUAD and control LUSC, with higher transcriptional diversity among the LUSC samples (Fig. 3b). T-LUSC showed extensive overlap with T-LUAD and partial overlap with both control LUAD and control LUSC. This suggests that T-LUSC retain some transcriptomic features of LUAD (Fig. 3b). PLSDA of genome-wide methylation showed substantial overlap between the profiles of T-LUAD and T-LUSC, with both of these groups more closely associated with control LUAD than with control LUSC (Fig. 3c). These data suggest that tumors undergoing LUSC transdifferentiation retain epigenomic features of their previous LUAD state.

### Identification of pathways dysregulated in LUSC transdifferentiation

To analyze signaling pathways dysregulated upon transdifferentiation, we performed differential gene expression and pathway enrichment analyses (GSEA) of T-LUSC versus T-LUAD samples (Fig. 3d and Additional file 1: Table S4). As expected, we observed upregulation of *TP63*, the gene encoding the squamous marker P40 in T-LUSC, which was further enriched in control LUSC (Additional file 2: Figure S2B). GSEA demonstrated downregulation of multiple immune response pathways in T-LUSC relative to T-LUAD, including neutrophil degranulation, interferon signaling, cytokine signaling, antigen presentation and complement activation. Together, these data suggest suppressed anti-tumor immune response in T-LUSC. We further observed enriched expression of genes (1) associated with a basal phenotype; (2) involved in cell cycle and DNA repair; (3) involved in epigenetic regulation (including EZH2, previously implicated in lineage plasticity [43, 44]) (Additional file 2**:** Figure S2C); (4) metastasis, a signature previously implicated in histologic transformation [43, 44]; and (5) genes involved in a variety of oncogenic signaling pathways, including MYC, NOTCH, Hedgehog, PI3K/AKT and WNT (Fig. 3d).

Consistent with these results, phospho-kinome arrays on microdissected T-LUAD (*N* = 5) and T-LUSC (*N* = 3) (Additional file 1: Table S4) further suggested T-LUSC-specific activation of DNA damage response, as indicated by increased CHK-2_T68 (Fig. 3e); of PI3K/AKT signaling, as suggested by increased AKT1/2/3_S473 and T308, PRAS40_T246, PYK2_Y402 and RSK1/2/3_ S380/386/377; and of WNT signaling (beta-catenin upregulation) (Fig. 3e). Western blotting confirmed increased expression of beta-catenin, pAKT_S473 and pPRAS40_T246. MYC protein expression was elevated in T-LUSC relative to T-LUAD, as demonstrated both by Western blotting (Fig. 3f) and by immunohistochemistry (5 out of 8 samples) (Fig. 3g, Additional file 1: Table S4), consistent with the RNAseq data (Fig. 3d). These data point to a possible role of PI3K/AKT signaling and MYC in LUSC transformation.

### Methylation changes may facilitate a stem-like state in LUSC transdifferentiation

To identify particular epigenetic mechanisms that might promote lineage plasticity between LUAD and LUSC, we analyzed differential methylation of TF-binding motifs (Fig. 3h). Site-specific methylation can inhibit TF binding and affect regulation of target gene expression [45]. We found hypermethylation of binding motifs for GATA6 in T-LUSC relative to T-LUAD (*p*-value < 0.001, *q*-value = 0.022). GATA6 is a known lineage specification factor amplified in adenocarcinomas of the upper gastrointestinal tract, promoting adenocarcinoma but not squamous cell carcinoma survival in the esophagus [46], and whose loss induces a shift in pancreatic cancer from adenocarcinoma to squamous metabolic phenotype [47]. Conversely we observed hypomethylation of binding motifs for genes involved in EMT and stemness such as *SNAI2* (SLUG) (*p*-value < 0.001, *q*-value = 0.019) and MYB (*p*-value < 0.001, *q*-value = 0.019) [48, 49]. We also observed T-LUSC-specific demethylation of multiple lineage-determining transcription factors, including ASCL1 (*p*-value = 0.001, *q*-value = 0.081) [50]; FOXI1 (*p*-value = 0.010, *q*-value = 0.081) [51]; and GRHL2 (*p*-value = 0.010, *q*-value = 0.109) [52, 53] (Fig. 3h). Our results suggest that site-specific methylation changes may promote lineage plasticity through altered binding of multiple lineage-determining transcription factors, facilitating lineage plasticity between LUAD and LUSC.

### Transcriptomic and methylomic alterations of transforming versus control tumors

**Fig. 4:**
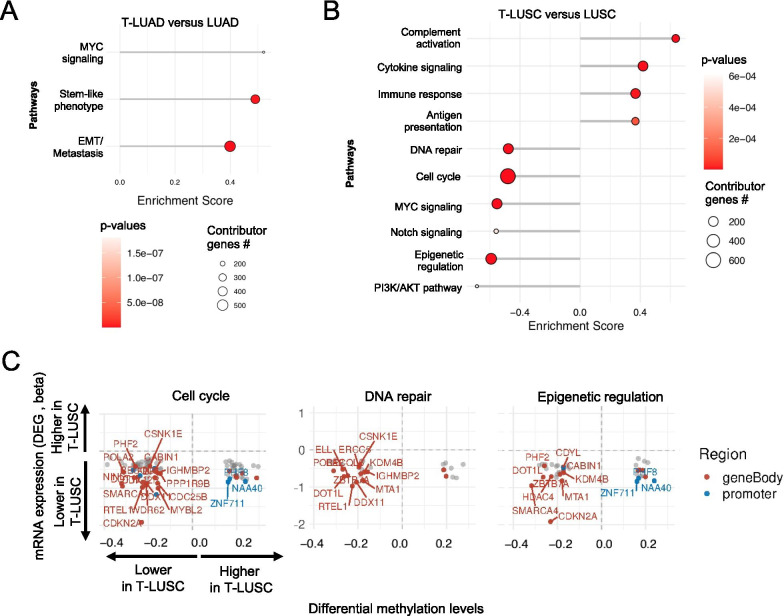
Integrative RNA and methylation analyses of T-LUAD and T-LUSC versus their control counterparts. **a** Pathway enrichment analyses on the DEGs of the T-LUAD versus LUAD comparison. **b** Pathway enrichment analyses on the DEGs of the T-LUSC versus de novo LUSC comparison. **c** Scatter plots showing differentially expressed genes exhibiting differential methylation levels in T-LUSC versus de novo LUSC comparison, grouped by pathways of interest. Significantly differentially expressed (*q* value < 0.05 and [beta] >  = log2(1.2)) and methylated (FDR < 0.05 and differential methylation level greater than 0.1) sites are highlighted. Those genes where increased gene body or promoter methylation is correlated with expression positively and negatively, respectively, are labeled

We next performed GSEA on differentially expressed genes in T-LUAD versus control LUAD to identify dysregulated pathways specific to LUAD at potential risk of transformation, which may comprise early events of LUSC transformation (Fig. 4a). We observed upregulation of genes involved in MYC signaling, stemness, and EMT/metastasis (Fig. 3d). However, transcriptomic dysregulation of these pathways was not driven by methylation events (Additional file 2: Figure S3A). Together, these support the occurrence a plastic phenotype in T-LUAD facilitating histological transdifferentiation.

We also performed GSEA on differentially expressed genes in T-LUSC versus control LUSC, to identify any molecular characteristic specific to LUSC that have undergone transdifferentiation. This analysis revealed higher immune activation in T-LUSC, including upregulation of gene pathways of complement activation, cytokine signaling, immune response and antigen presentation (Fig. 4b). This suggests a degree of immune inflammation in T-LUSC tumors relative to their de novo counterparts, consistent with enrichment for the secretory subtype characterized by high tumor-associated immune response [32]. T-LUSC demonstrated decreased expression relative to control LUSC of genes involved in cell cycle/DNA repair and epigenetic regulation pathways, which seemed to be driven by methylation changes (Fig. 4c). We also observed downregulation of genes involved in MYC, Notch and PI3K/AKT pathways (Fig. 4b), which seemed to be independent to methylation events (Additional File 2: Figure S3B). Interestingly, all these pathways were upregulated also in T-LUSC relative to T-LUAD (Fig. 3d).

### MYC overexpression and AKT overactivation induce expression of LUSC markers in LUAD preclinical models

Next, we sought to identify molecular drivers of LUSC transformation among the signaling pathways upregulated in the RNAseq/protein analyses. Among these, the PI3K/AKT pathway has been extensively involved in lineage plasticity [1]. LUAD undergoing histologic transformation are enriched for mutations in this pathway [2, 3, 54], and *Pten* loss promotes squamous tumors in an adenocarcinoma model of prostate cancer [55]. MYC signaling has also been similarly implicated as a driver of stemness and histological transdifferentiation [1, 43].

**Fig. 5:**
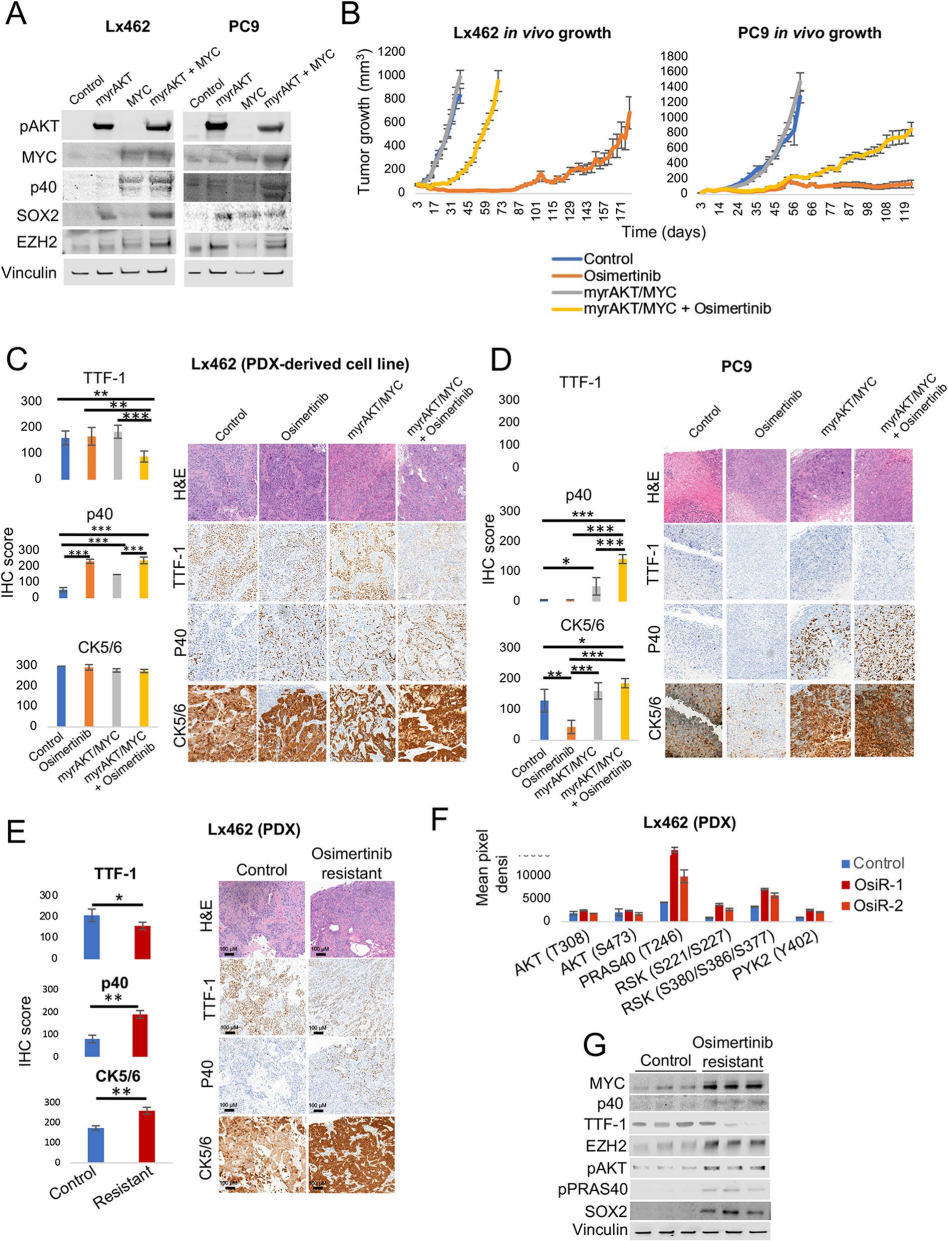
PI3K/AKT and MYC pathways induce a squamous-like phenotype in EGFR-mutant LUAD models. **a** Western blot showing the expression of the squamous marker P40, as well as of SOX2 and EZH2 in the EGFR-mutant LUAD PDX-derived cell line Lx462 and in the PC9 cell line, expressing exogenous myrAKT, MYC, or both. **b** Tumor growth on subcutaneous xenografts of the control and myrAKT/MYC-overexpressing conditions for both cell lines, untreated or treated with osimertinib (*N* = 5 mice/condition). Mean ± SEM tumor size is shown. Representative images for H&E, TTF-1, P40 and CK5/6 IHC stains and barplot showing IHC quantification (mean ± SEM score values per group are shown) of the Lx462 (**c**) and PC9 (**d**) cell line xenografts from the subcutaneous xenografts shown in (**b**). **e** Representative images for H&E, TTF-1, P40 and CK5/6 IHC stains and barplot showing IHC quantification (mean ± SEM score values per group are shown) of the control and osimertinib-resistant Lx462 PDXs. **f** Bar plot showing differential phosphorylation of genes involved in the AKT pathway, as determined by an antibody array on one control and two osimertinib-resistant Lx462 PDX derivatives (OsiR-1 and OsiR-2). **g** Western blot assessment of the expression of MYC, P40, TTF-1, EZH2, pAKT, pPRAS40 and SOX2 in control and osimertinib-resistant Lx462 PDXs. *p*-values legend: *p < 0.05, **p < 0.01, ***p < 0.001

To test the role of these factors in promoting squamous transformation of LUAD, we first overexpressed MYC, a constitutively active isoform of AKT (myrAKT), or their combination, in a short-term cultured LUAD PDX-derived cell line (LX462) and in a LUAD cell line (PC9) (Fig. 5a). Both models harbor an *EGFR* mutation, modeling a setting where LUSC transformation has been extensively described [4]. In vitro we observed that combined MYC/myrAKT overexpression induced the expression of the squamous marker P40 in both cell lines, with MYC overexpression alone inducing this at lower levels in one of the cell lines under study (Lx462). Interestingly, combined MYC/myrAKT overexpression also induced the expression of EZH2 and of SOX2 (Fig. 5a), the latter being a known driver of LUSC [56, 57].

Next we wanted to assess the effect modulating these factors on tumor growth. We performed subcutaneous injection of control and MYC/myrAKT-overexpressing Lx462 and PC9 cell lines in the flank of NSG mice. We did not observe any appreciable difference in tumor growth in untreated tumors. In mice treated with osimertinib, MYC/myrAKT markedly accelerated acquired treatment resistance in both tumor models (Fig. 5b).

Although the xenografts did not fully transdifferentiate to classical squamous morphology with intercellular bridges and heavy keratinization, IHC analysis of the LUAD marker TTF-1 and of the LUSC markers p40 and CK5/6 confirmed increased expression of p40 in the untreated MYC/myrAKT-overexpressing tumors versus the control condition in both models, consistent with our in vitro results (Fig. 5c,d). We observed further p40 induction in the osimertinib-treated MYC/myrAKT-overexpressing tumors again in both models. In PC9, but not LX462, we observed increased CK5/6 levels in the MYC/myrAKT-overexpressing tumors (either untreated or treated with osimertinib) relative to control tumors without MYC/myrAKT. Notably, in the LX462 model, TTF-1 expression was downregulated only in the osimertinib-treated MYC/myrAKT condition (Fig. 5c), further supporting the potential for EGFR inhibition to facilitate transdifferentiation to a squamous-like phenotype. No TTF-1 expression was observed in the PC9 model tumors (Fig. 5d). These results suggest that combined MYC overexpression and AKT overactivation can promote a squamous-like phenotype similar to that reported for the secretory squamous subtype [32] (i.e., partially retained TTF-1 expression, moderate and non-ubiquous p40 expression) which is further accentuated by EGFR inhibition.

Expression of p40 was increased in the osimertinib-treated control condition in the Lx462 cell line xenografts (Fig. 5c), suggesting that this model may be prone to spontaneous potentiation of squamous features under selective pressure of EGFR-targeted therapy, even in the absence of exogenous AKT and MYC signaling. We confirmed this by the generation of osimertinib-resistant Lx462 PDXs. Comparison of control untreated versus osimertinib-relapsed Lx462 tumors showed increased p40 expression after osimertinib relapse (Fig. 5e). We further observed decreased TTF-1 expression and increased CK5/6 expression in the osimertinib-relapsed Lx462 PDX tumors (Fig. 5e), as well as increased *KRT5* and decreased *AGR2* mRNA expression (Additional File 2: Figure S4A), a recently described qualitative signature for LUSC tumors(58). Additionally, performance of pathway enrichment analysis on DEGs in osimertinib-relapsed versus control Lx462 (Additional File 2: Figure S4B) showed high consistency with the pathways we found dysregulated in transforming clinical samples (Fig. 3d), further supporting the acquisition of a LUSC phenotype during acquisition of osimertinib resistance. Genetic relatedness of control and osimertinib-resistant tumors was confirmed by MSK-IMPACT [59], which shared the *EGFR* mutation, among others.

Analysis by phospho-kinase array suggested activation of the AKT signaling pathway including phosphorylation of downstream PRAS40 in osimertinib-relapsed LX462 PDXs (Fig. 5f). PRAS40 phosphorylation occurred in conjunction with upregulation of EZH2, SOX2 and MYC in the osimertinib-relapsed LX462 PDXs (Fig. 5g), largely recapitulating the pathways activated by exogenous myrAKT and MYC and upon LUSC transformation in our clinical samples. Taken together, these data support the hypothesized involvement of MYC and AKT signaling in the induction of a marker expression profile more compatible with LUSC.

### EZH2 or AKT inhibition may interfere with osimertinib “squamous-like” relapse

**Fig. 6:**
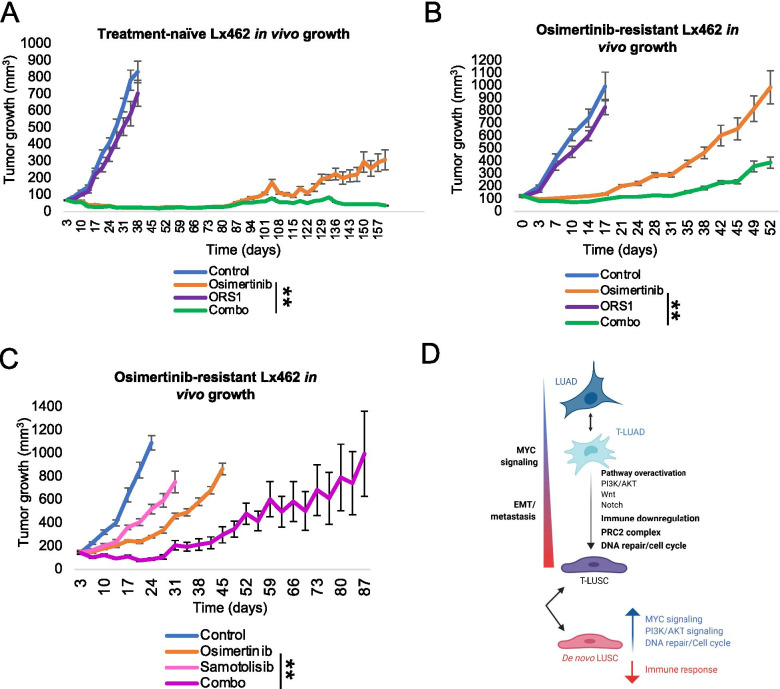
PI3K/AKT pathway and EZH2 as therapeutic targets for LUSC transformation. **a** In vivo tumor growth of the LUAD EGFR-mutant PDX model Lx462 with the EGFR inhibitor osimertinib, the EZH1/2 inhibitor ORS1, or their combination (*N* = 5 mice/treatment group). In vivo tumor growth of the squamous-like osimertinib-resistant EGFR-mutant PDX model Lx462 with the EGFR inhibitor osimertinib, the EZH1/2 inhibitor ORS1, or their combination (*N* = 6 mice/treatment group) (**b**); or with osimertinib, the AKT inhibitor samotolisib or their combination (*N* = 6 mice/treatment group) (**c**). For in vivo tumor growth, group mean tumor size ± SEM is shown. Statistical differences in tumor sizes were assessed by a two-tailed Student´s *t*-test, using the tumor sizes for osimertinib-treated group experiment endpoint. **d** Schematic summarizing the phenotypes and pathways altered upon LUSC transformation

We next sought to explore how inhibition of targets found upregulated in our clinical and preclinical analyses may interfere with osimertinib sensitivity and with the acquisition of the “squamous-like” phenotype observed in our Lx462 PDX model after osimertinib relapse. We first focused on EZH2, the enzymatic subunit of the PRC2 complex, which has been previously defined as a mediator of MYC-induced stemness [60] and as a factor promoting histological transformation [43, 44]; it was also notably upregulated in our analysis of clinical biospecimens (Fig. 5g and Additional file 2: Figure S2C). We treated the Lx462 PDX with osimertinib, the EZH1/2 inhibitor ORS1, or their combination (Fig. 6a). While ORS1 alone had no significant effect on tumor growth, ORS1 was able to prevent acquired resistance to osimertinib in Lx462 tumors (Fig. 6a and Additional file 2: Figure S4C). To assess whether this strategy could also be used to resensitize to EGFR inhibition after acquisition of the “squamous-like” phenotype, we tested the efficacy of osimertinib plus ORS1 in osimertinib-relapsed (“squamous-like”) Lx462 tumors. These tumors also demonstrated no significant response to ORS1 alone, and early progression on osimertinib (Fig. 6b). ORS1 treatment again potentiated the anti-tumor efficacy of osimertinib (Fig. 6b and Additional file 2: Figure S4D), demonstrating over 60% tumor growth inhibition (TGI) relative to the osimertinib-treated group at experiment endpoint (*p* = 0.008). We assessed markers of LUAD and LUSC in the tumors taken down at study endpoint; while there was a slight increase in TTF-1 expression in the combination group compared to the osimertinib group, no differences in P40 or CK5/6 staining was observed among different study cohorts: neither EZH2 inhibition alone nor the combination reverted tumors back to pre-transformation P40 levels (Additional file 2: Figure S4E).

Finally, our clinical and preclinical data also suggested a role for PI3K/AKT pathway activity as a driver of LUSC transdifferentiation. We therefore similarly assessed the efficacy of the AKT inhibitor samotolisib in the osimertinib-relapsed “squamous-like” Lx462 PDX tumors (Fig. 6c). Samotolisib also enhanced the efficacy of osimertinib in this model, resulting in a TGI of 65% relative to osimertinib alone at osimertinib group endpoint (*p* = 0.002, Fig. 6c). No increased toxicity was observed with the combination treatment relative to osimertinib single agent, as assessed by body weight (Additional file 2: Figure S4F). Immunohistochemistry for lineage markers showed no differences in TTF-1, P40 or CK5/6 expression (Additional file 2: Figure S4G).

In summary, although these results would need to be validated in additional fully LUSC-transformed models, these data nominate EZH2 inhibition as a potential therapeutic target in this setting that may enhance the durability of osimertinib response in tumors at risk of lineage transformation. Our results also suggest that inhibition of EZH2 or AKT, even if not able to revert the osimertib-induced “squamous-like” phenotype, may be able to re-sensitize *EGFR*-mutant tumors to osimertinib after its acquisition.

## Discussion

Lineage plasticity is a key source of tumor adaptability to adverse conditions or selective pressures and is increasingly recognized as a driver of therapeutic resistance [1]. The first described examples of this in LUAD were cases of neuroendocrine transformation to a tumor resembling small cell lung cancer [2, 3]. With the advent of increasingly potent and selective targeted therapies addressing “on-target” resistance, we may see increasingly prevalent, and increasingly diverse, examples of tumor escape by histologic transformation. Transformation of LUAD to LUSC as a mechanism of acquired resistance has only recently been described [4] and has been less extensively characterized than neuroendocrine transformation. *EGFR*-mutant LUAD progressing through squamous transformation has a poor prognosis [4], as does primary mixed histology LUAD/LUSC [9]. Here we provide the first comprehensive characterization of squamous transformation, through a multi-omic analysis of a cohort of mixed LUAD/LUSC tumors as well as isolated pre- and post-LUAD to LUSC transformation cases.

Our study was predicated on the hypothesis that clonally related mixed histology tumors, in which both LUAD and LUSC lineages are in temporal and spatial proximity, might provide general insight into relevant biologic pathways dictating differentiation between these lineages, and could identify therapeutic targets of relevance to squamous transdifferentiation. A limitation of this study is the modest number of cases available of mixed histology tumors in which clean microdissection could be performed. This analysis provided a progressive signaling landscape of LUAD to LUSC transformation, and identified several potential targets including the PI3K/AKT and MYC activation, and PRC2 complex action (Fig. 6d). Validating our approach, concomitant activation of AKT and MYC in LUAD preclinical models induced LUSC features, with further augmentation under the selective pressure of a driver oncogene-targeted therapy. These results suggested that even if targeted therapy may be accelerating transdifferentiation, selective pressure by treatment may not be essential for it to occur, which was consistent with the fact that the majority of adenosquamous samples under analysis had not received any treatment before collection.

Confirming the potential translational relevance of these findings, targeted inhibition of EZH2 prevented emergence of acquired osimertinib resistance in an EGFR-mutant LUAD prone to lineage plasticity and partially restored osimertinib efficacy in the osimertinib-resistant derivative with increased squamous features. AKT inhibition demonstrated similar combinatorial efficacy in this context. Even if these results would need to be validated in additional models exhibiting full squamous-transformation, these findings nominate targets with a potential translational relevance, as multiple pharmacologic inhibitors of both of these pathways are clinically available.

The cell of origin of adenosquamous tumors has not been clearly defined, and it is possible that multiple cell types could give rise to such tumors. Our genomic data confirm prior findings that LUAD and LUSC components of adenosquamous carcinomas share a clonal origin. Our epigenomic and transcriptomic data indicate that T-LUSC exhibits a molecular profile close to that of LUAD, but distinct from that of de novo LUSC. These results support a role for lineage plasticity in their development, rather than colocalization of independent tumors, as previously suggested [7, 36]. The directionality of transformation in adenosquamous cases cannot be assumed, and it is possible that both histologies arise in parallel establishing a cooperative relationship, as has been previously reported for other tumor types in which distinct intratumoral subpopulations cooperate to promote therapeutic resistance or metastasis [61].

While apparent enrichment of *TBX3, MET* and *RBM10* mutations in the T-LUAD relative to control LUAD may provide a genetic context favoring lineage plasticity and histological transdifferentiation, the absence of recurrent genomic alterations distinguishing paired LUAD and LUSC components suggests that these or other genomic alterations are not driving this process, which appears to be rather transcriptionally driven. The commonalities among altered transcriptional programs between paired T-LUAD and T-LUSC, and the focal methylation changes affecting binding of known transcriptional regulators implicated in stemness and plasticity, strongly underscore the primary epigenetic nature of lineage plasticity.

Cross-comparison of the data reported here with prior analyses of histologic transformation in other contexts reveals interesting commonalities and distinctions—reflecting pathways that may facilitate lineage plasticity per se, and pathways that may in particular drive conversion toward particular alternative lineages. We would anticipate that shared promoters of lineage plasticity might include factors required for maintaining stem or progenitor capacity in embryonic and fetal development: for example, as noted here, MYC, EZH2 (and PRC2 complex activity generally), GATA proteins, SLUG, MYB, WNT, Hedgehog and Notch signaling. Indeed, several of these, most notably MYC and PRC2 activity, have been implicated across multiple prior studies, including in of lung and prostate adenocarcinomas to aggressive small cell neuroendocrine tumors [1, 10, 33, 35]. PI3K/AKT pathway activity and drivers of EMT while not canonical stem cell pathways have also been identified in neuroendocrine transformation [1, 4, 35, 52]. In contrast, a canonical requirement of neuroendocrine transformation in both prostate and lung cancer, concomitant loss or mutational inactivation of both TP53 and RB1, does not appear to be a feature of squamous transformation (Fig. 2b).

## Conclusions

The study of plasticity mechanisms leading to histological transformation and eventually therapy resistance is key to understand tumor evolution and to design strategies to manage patients with T-LUSC. Our data point in particular to AKT, MYC, and PRC2 complex signaling as playing key roles in this histologic transformation. Using preclinical models of *EGFR*-mutant LUAD, we observed that the combination of AKT stabilization and MYC overexpression induced a “squamous-like” phenotype, which was further accentuated by osimertinib treatment. Pharmacologic inhibition of either AKT or PRC2 complex activity substantially augmented the efficacy of osimertinib treatment in a PDX model acquiring squamous features under EGFR inhibition. Here, we provide the first comprehensive molecular characterization of LUSC transdifferentiation and suggest potential drivers and therapeutic approaches for these tumors.

## Supplementary Information

**Additional file 1**: **Table S1**. Clinical characteristics of the adenosquamous and pre /post-transformation tumors under study. **Table S2**. Clinical characteristics of the de novo LUSC tumors under study. **Table S3**. Clinical characteristics of the control LUAD tumors under study. **Table S4**. Summary of molecular applications per sample.

**Additional file 2**: Supporting supplementary figures.

## Data Availability

The datasets used and/or analyzed during the current study, as well as all materials used, are available from the corresponding author on reasonable reques. RNAseq data are available in https://drive.google.com/drive/folders/1YX6jNesWy_rsdx46eErkc_bHEymqNVia?usp=sharing (data will be deposited in a public repository upon acceptance).

## Abbreviations

LUAD: Lung adenocarcinoma
LUSC: Lung squamous carcinoma
IHC: Immunohistochemistry
PDX: Patient-derived xenograft
GSEA: Gene set enrichment analysis
TPM: Transcripts per million
