# Supplementary material for: Comprehensive molecular characterization of lung tumors implicates AKT and MYC signaling in adenocarcinoma to squamous cell transdifferentiation
Source: J Hematol Oncol. 2021 Oct 16;14:170. doi: 10.1186/s13045-021-01186-z (PMC8520275; doi:10.1186/s13045-021-01186-z)
Supplement: Supplementary file 2 — Additional file 2: Supporting supplementary figures. [file 13045_2021_1186_MOESM2_ESM.pdf]

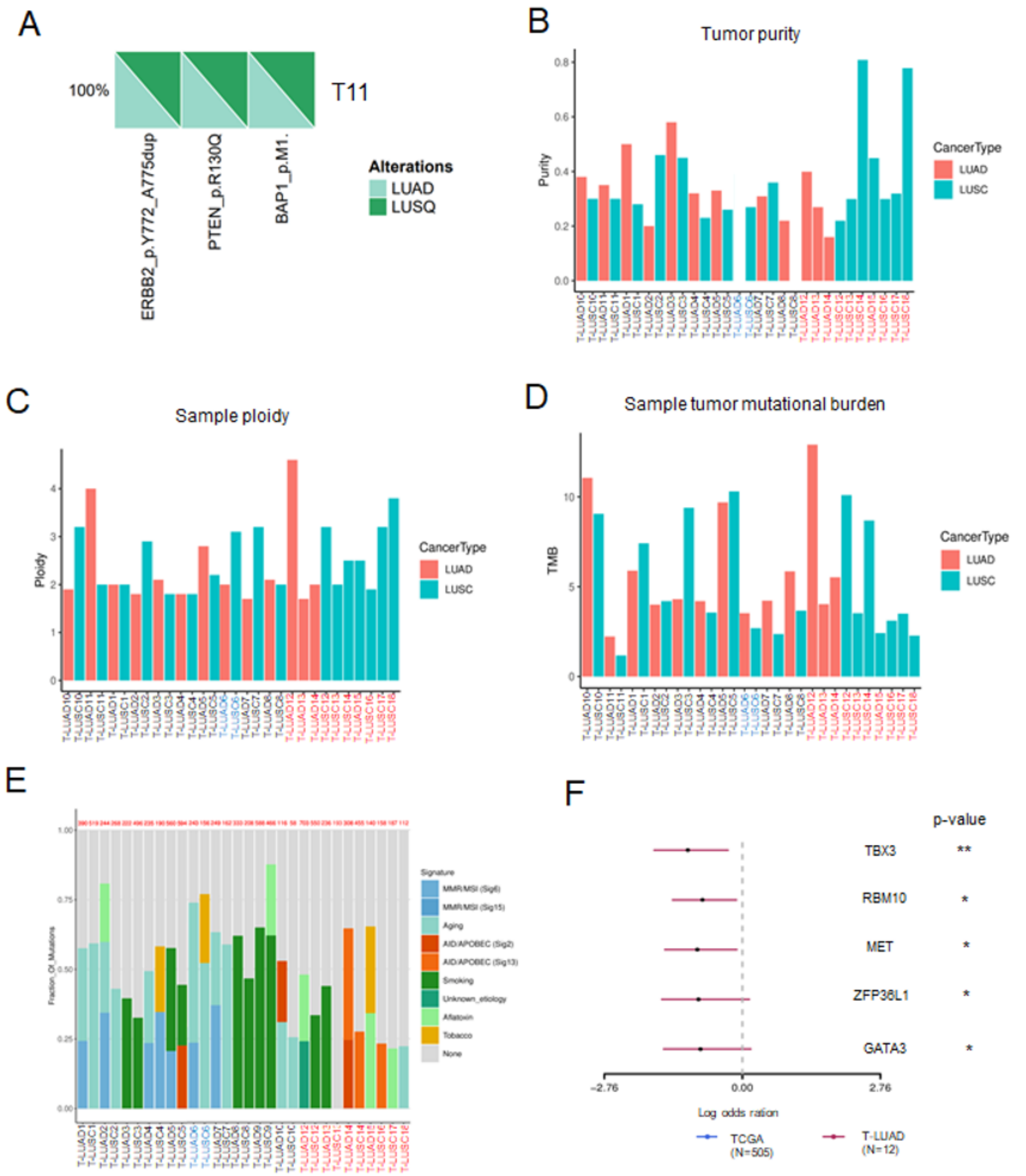

Supplementary Figure S1. Related to Figure 2. (A) Common mutations in the LUAD and LUSC components of the mixed LUAD/LUSC case T11. Bar plots showing tumor purity (B), ploidy (C) and mutational burden (D) of samples in the cohort analyzed by WES. Samples for which purity is not shown exhibited low tumor content, preventing the analysis pipeline to provide an estimate of tumor purity. (E) Enrichment in mutational signatures on the samples in our cohort analyzed by WES. (F) Plot showing enrichment in mutations (shown as odds ratio) in the T-LUAD samples versus TCGA LUAD. p-values legend: * p<0.05, **p<0.01. Samples IDs in black indicate that they come from a combined histology specimen where LUAD and LUSC components are genetically related. Samples IDs in blue indicate that they come from a combined histology specimen where LUAD and LUSC components are genetically unrelated. Samples IDs in red indicate that they come from a pre-/post-transformation specimen.

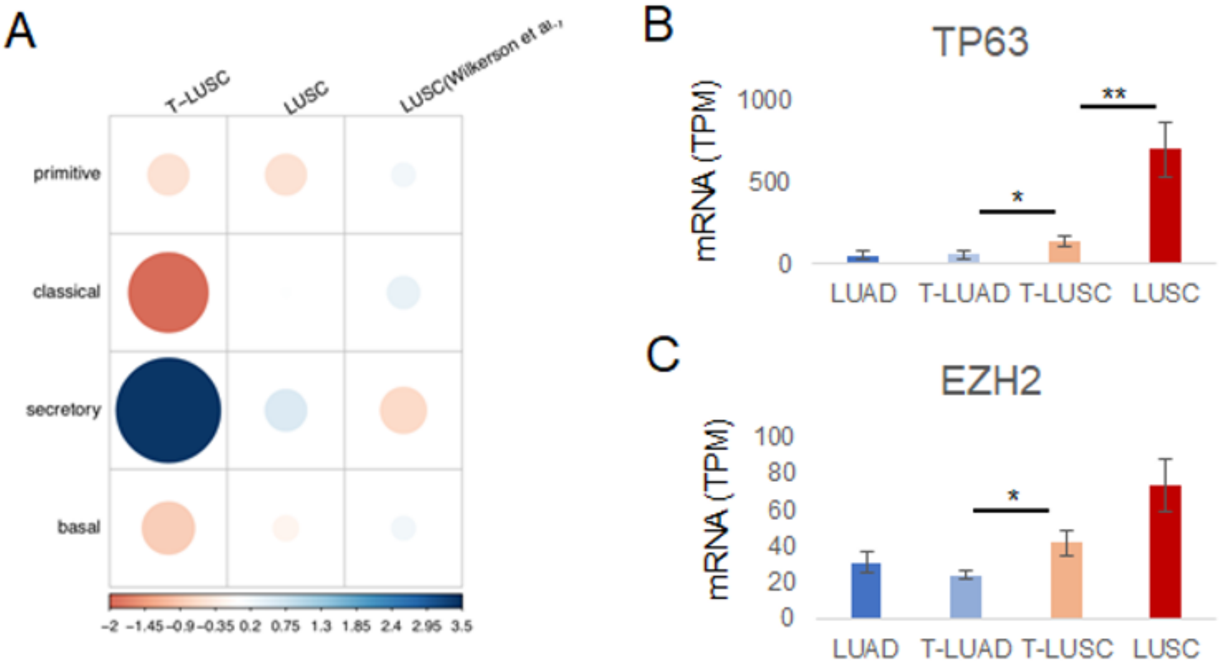

Supplementary Figure S2. Related to Figure 3. (A) Dot plot showing the correlation of the different LUSC subtypes with the cohorts under study. mRNA expression levels of TP63 (B) and EZH2 (C) in LUAD, T-LUAD, T-LUSC and LUSC samples in our cohort. Expression level differences were assessed by performing a two-tailed Student's t-test. p-values legend: * $p<0.05$, ** $p<0.01$

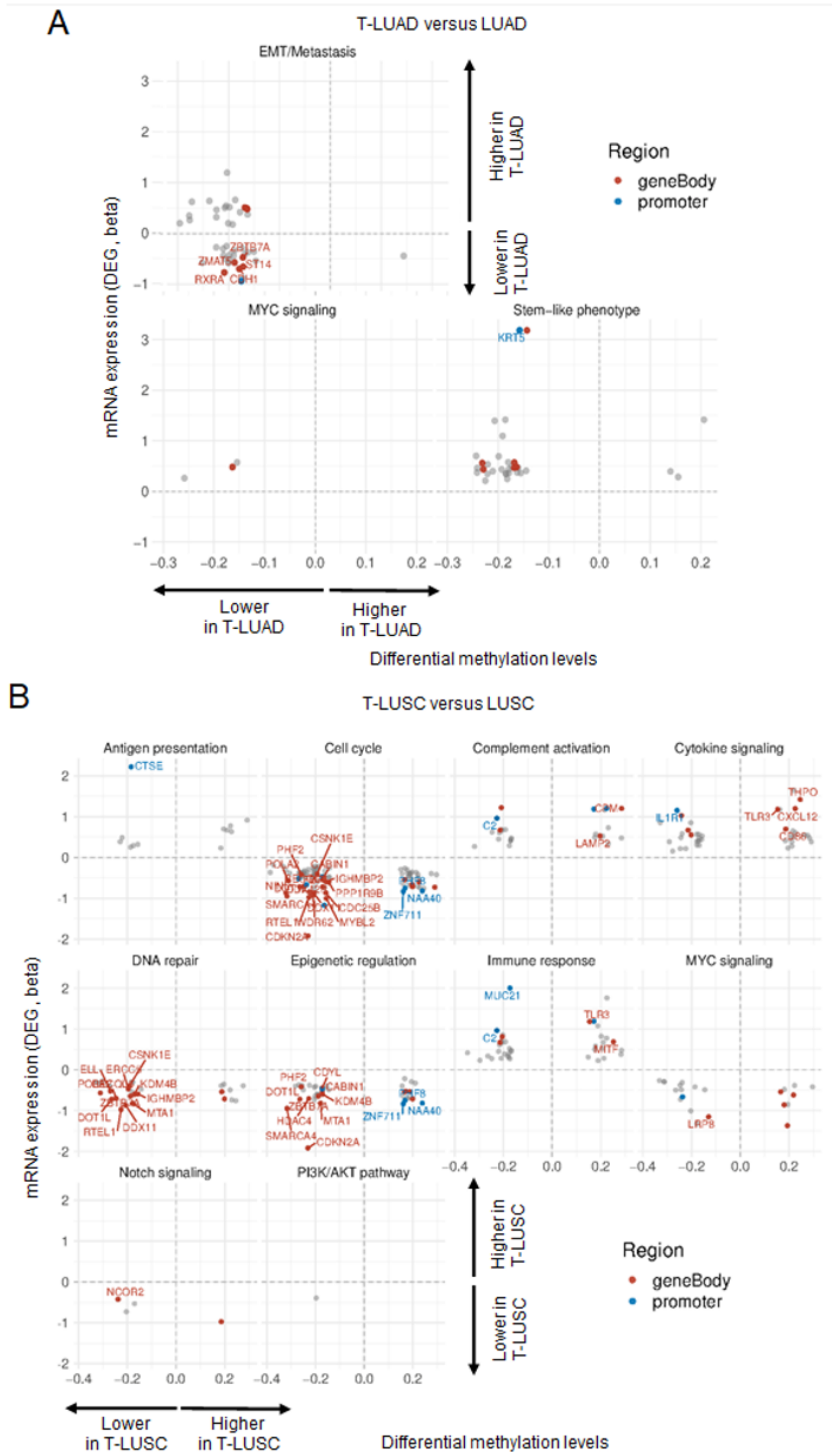

Supplementary Figure S3. Related to Figure 5. Scatter plots showing differentially expressed genes exhibiting differential methylation levels in T-LUAD versus control LUAD (A), or T-LUSC versus control LUSC (B) comparisons, grouped by pathways of interest. Significantly differentially expressed (q value < 0.05 and [beta] >= log2(1.2)) and methylated (FDR < 0.05 and differential methylation level greater than 0.1) sites are highlighted. Those genes where increased gene body or promoter methylation is correlated to expression positively and negatively, respectively, are labeled.

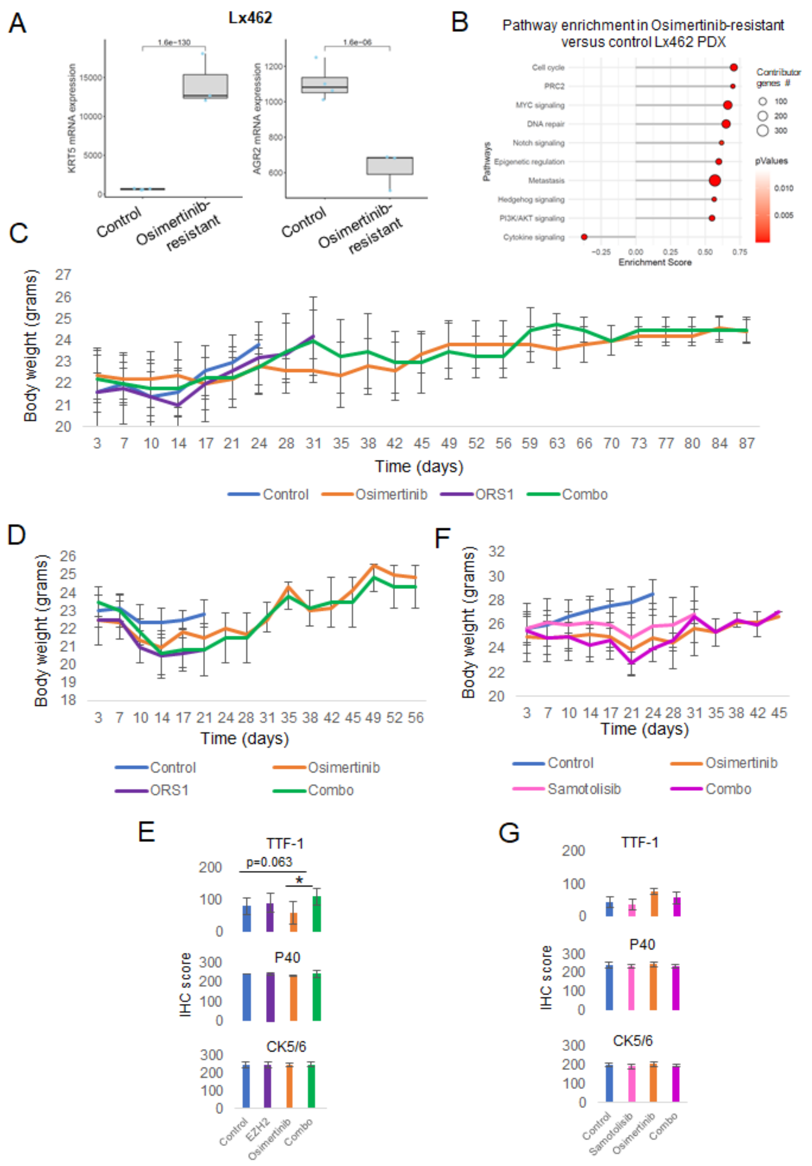

Supplementary Figure S4

Supplementary Figure S4. Related to Figure 7. (A) KRT5 and AGR2 expression on control and osimertinib-resistant (squamous-like) Lx462 PDX. (B) Pathway enrichment analyses on DEGs for osimertinib-resistant (squamous-like) versus control Lx462 PDX showing common dysregulated pathways with those on transforming clinical samples (see **Figure 3D**). Mouse body weight measurements of the mice bearing the treatment-naïve (C) or osimertinib-resistant (D) Lx462 PDX tumors treated with osimertinib, ORS1 or their combination. (E) Barplots showing IHC quantification of TTF-1, P40 and CK5/6 stains in the control and osimertinib-, ORS1- and combination-treated groups (mean ± SEM score values per group are shown). (F) Mouse body weight measurements of the mice treated with osimertinib, samotolisib and their combination. (G) Barplots showing IHC quantification of TTF-1, P40 and CK5/6 stains in the control and osimertinib-, samotolisib- and combination-treated groups (mean ± SEM score values per group are shown). Expression level differences were assessed by performing a two-tailed Student's t-test. p-values legend: * $p<0.05$.
